# Social Networking Smartphone Applications and Sexual Health Outcomes among Men Who Have Sex with Men

**DOI:** 10.1371/journal.pone.0086603

**Published:** 2014-01-23

**Authors:** Justin J. Lehmiller, Michael Ioerger

**Affiliations:** 1 Department of Psychology, Harvard University, Cambridge, Massachusetts, United States of America; 2 Department of Psychology, Syracuse University, Syracuse, New York, United States of America; UNC Project-China, China

## Abstract

Several smartphone applications (apps) designed to help men who have sex with men (MSM) find casual sexual partners have appeared on the market recently. Apps of this nature have the potential to impact sexual health and behavior by providing constant access to a large supply of available partners. In this study, the sexual health history, behavior, and personality of MSM who use these apps was compared to MSM who meet partners in other ways. A sample of 110 adult MSM was recruited online to complete a cross-sectional survey. All participants were either single or involved in a non-exclusive romantic relationship. There were no statistically significant differences between app users and non-users in frequency of insertive or receptive anal sex without a condom. However, app users reported significantly more sexual partners and had a higher prevalence of ever being diagnosed with an STI than did non-users. App users did not differ from non-users on any demographic or personality variables (including erotophilia, sensation seeking, and self-control); however, when adjusting lifetime total sex partners for those met specifically through apps, app users still had significantly more partners. This pattern of results suggests that app users may be more sexually active in general. More work is needed to fully understand the association between this emerging technology and potential sexual health risks.

## Introduction

Smartphone applications (apps) designed to help men who have sex with men (MSM) find casual sexual partners have inundated the worldwide app market in recent years. These apps include Grindr, FindFred, Growlr, Scruff, and many others, each with some variation in specific focus and target audience. Perhaps the most popular of these is Grindr, a geosocial networking app designed to let “gay, bi, and curious guys” find other nearby men by showing thumbnail photos of other users arranged from closest to farthest away. Grindr, which debuted in 2009 and currently boasts over four million users, allows members to chat, share photos, and (if desired) send their exact location. Users can also enable the app to send instant notifications of messages from prospective partners, effectively allowing people to arrange sexual encounters even when they are not actively looking. Although Grindr and other such apps are officially advertised as offering social networking and dating services, MSM who use these apps frequently report using them to find sexual partners [1].

By providing constant access to a steady stream of available partners, smartphone apps of this nature have the potential to impact sexual health and behavior in many ways; however, research has yet to explore whether and how such apps are even linked to the sexual practices of MSM. The goals of the present study were therefore (1) to obtain descriptive information on MSM who seek sexual partners via smartphone apps and (2) to compare the sexual health histories of app users and non-users. We also sought to compare the demographic and psychological profiles of app users and non-users to determine whether these apps attract persons who are drawn to greater sexual risk.

### Technology and Sexual Risk-Taking

Ever since MSM began seeking sex over the Internet, scientists and public health officials have warned of the dangers of this method of meeting partners due to the speed with which anonymous sexual encounters can be arranged. These warnings have seemingly been validated by research demonstrating that online sex seeking is associated with more risks than casual sex arranged in-person. For instance, online partnering has been linked to reporting greater numbers of sexual partners [2], [3], [4], a higher likelihood of practicing unprotected anal intercourse (UAI) [2], [5], and a higher probability of having ever been diagnosed with a sexually transmitted infection (STI) [3], [5], [6]. Although some conflicting findings have emerged, meta-analyses have established that arranging casual sex online is linked to greater risk relative to meeting partners offline [7].

Given these findings, one might expect that sex seeking via smartphone apps would be associated with heightened sexual risk as well. However, these apps could potentially produce risks that are even *greater* than previously observed with computer-based websites, given that people tend to carry their smartphones with them at all times. In addition, these apps can be enabled to notify users instantaneously when they are being sought by others. The location-based nature of some of these apps could also potentially promote faster partnering by narrowing the search field to those who are already nearby.

That said, there has been longstanding controversy over whether technologies such as this promote riskier sexual behavior (known as the “accentuation hypothesis”), or whether people who practice riskier behavior are drawn to the technology (known as the “self-selection hypothesis”) [7]. Recent research on Internet sex-seeking behavior has provided support for the self-selection hypothesis by indicating that many MSM who seek partners online are also seeking partners offline, and these MSM report more offline partners than MSM who *only* meet partners offline [9]. This suggests that MSM who use the Internet for casual sex may be seeking a greater numbers of partners in general. This research also revealed that utilizing both online and offline methods was associated with greater risk compared to online-only and offline-only methods.

### Personality and Sexual Risk-Taking

If persons who utilize technology to facilitate casual sex differ from those who meet in other ways (e.g., face-to-face), what accounts for that difference? One possibility is differences in personality, given that many facets of personality have previously been linked to sexual risk-taking. In the present study, we consider three relevant individual difference variables that might affect one’s interest in utilizing smartphone apps to facilitate casual sex: sensation seeking, erotophilia, and self-control.

#### Sensation seeking

Sensation seeking refers to a desire to partake in activities that are stimulating. In the context of sexual behavior, sensation seeking is closely associated with searching out sexual stimuli that are novel and exciting [10]. This drive for thrilling, new sexual experiences is associated with risky sexual behavior, with research finding that higher scores on measures of sexual sensation seeking are associated with higher rates of unprotected sex [11], [12], a greater number of sexual partners [10], [13], and a higher likelihood of being HIV-positive [12]. Sexual sensation seeking has also been identified as a moderator between alcohol and drug use prior to sex and higher rates of UAI for MSM [14]. This personality variable has also been identified as a moderator between Internet use and sexual risk taking behaviors among MSM [15].

#### Erotophilia

Erotophilia is the level of positive affect a person has for sex-related behavior, media, and thoughts [16]. It is typically assessed on a continuous scale, which ranges from erotophobic to erotophilic. Higher scores (i.e., greater erotophilia) are associated with having stronger, more favorable attitudes toward sexual cues. Additionally, there is a positive correlation between erotophilia and risky sexual behavior, such that more erotophilic persons express greater willingness to have casual sex and report higher numbers of sexual partners [17], [18].

#### Self-control

Self-control is a person’s ability to self-regulate their behaviors by overriding or inhibiting competing urges or desires. Capacity for exerting self-control is an individual difference that varies from person to person (i.e., trait self-control); however, our self-control abilities can also fluctuate to some degree across situations (i.e., state self-control) [19]. In the realm of sexual behavior, people with lower trait self-control engage in riskier sexual behaviors than persons with a higher capacity for self-control. Sexual health risks associated with lower self-control include having higher numbers of sexual partners, a greater likelihood of having unprotected sex, and having been diagnosed with an STI [20], [21], [22]. Lower levels of trait self-control are also associated with having a more difficult time staying faithful to one’s romantic partner [23].

We tentatively expected that sensation seeking, erotophilia, and self-control would all be associated with use of smartphone apps that facilitate locating casual sex partners. These apps would likely be appealing to individuals who (1) seek novelty and excitement, (2) have positive attitudes toward sex, and/or (3) have a more difficult time controlling sexual urges and impulses.

### The Present Research

We conducted an Internet-based study to learn more about the sex lives of MSM who meet sexual partners via smartphone apps and also to compare the sexual health histories and personalities of app users and non-users. Online data collection was pursued over college student subject pool recruitment so as to provide greater demographic diversity. We advanced the following hypotheses.

First, consistent with previous research linking online sex seeking to greater risk-taking [7], we predicted that app users would report higher numbers of recent sexual partners, more instances of recent UAI, and more reports of previous STI diagnoses compared to non-users. Second, to the extent that the self-selection hypothesis is true, we predicted that app users would report having a significantly greater number of lifetime sexual partners than non-users and that, even when the number of partners met through smartphone apps is subtracted from app users’ lifetime total, app users should still report having had more partners. Finally, and also consistent with the self-selection hypothesis, we expected that any differences in sexual risk behavior would be explained by psychological differences between app users and non-users. In other words, the link between app use and risky sex should be mediated by heightened levels of sensation seeking or erotophilia, and/or lower levels of self-control.

## Materials and Methods

### Participants and Ethics Statement

Participants were recruited for an Internet study of “men’s sexual attitudes and behaviors” for which the only selection criteria were (1) being age 18 or older and (2) being a man who has previously had sex with at least one other man. The Harvard University Committee on the Use of Human Subjects approved the protocol, and the data reported in this paper can be obtained from the first author upon request. Participants were required to provide informed consent via a consent button on the first page of the survey. No compensation was offered for taking part in this study.

### Materials and Procedure

Participants were recruited via solicitation notices posted on various Facebook and Twitter feeds for sexuality interest groups, as well as several LGBT student center listservs at U.S. colleges and universities. Notices were sent to schools in a broad cross-section of states in order to obtain geographic diversity. Aside from a few participants who referred friends or colleagues via email or social media, there were no other significant recruitment sources. Participants were not told that this was a study of social networking smartphone applications so as not to induce further selection bias.

For participants who advanced to the questionnaire website, informed consent was taken via a consent button. Upon providing consent, participants completed the measures listed below. For participants who indicated that they had a current account with a relevant smartphone application, they first completed a series of questions about their usage of this app; non-users skipped these questions entirely. Upon completing the survey, all participants were directed to an online debriefing page.

App users were asked about the specific application(s) they currently utilize for meeting sexual partners via their smartphone. In addition, they were asked to indicate the number of times per day they open or log onto the app, approximately how many minutes they spend engaged with the app in pursuit of sexual partners during each session (e.g., chatting, viewing photos or profiles), and whether they have the app enabled to send “push notifications” (i.e., immediate alerts regarding events that have occurred within the app, such as an incoming message from another user). App users were also asked about the specific number of oral and anal sex partners met through these apps, as well as whether any of these sexual partners eventually turned into romantic partners.

Additionally, all participants (both app users and non-users) completed a battery of measures about their personality and sexual practices. Three personality measures were administered: sensation seeking, erotophilia, and self-control. Each of these measures was rated on a 9-point scale ranging from 1 (*do not agree at all*) to 9 (*agree completely*). The 4-item Brief Sensation Seeking Scale (BSSS) [24] was administered, which included items such as “I prefer friends who are exciting and unpredictable” and “I like to do frightening things.” Although the BSSS does not directly assess *sexual* sensation seeking, the BSSS and sexual sensation seeking are highly correlated [9]. To measure erotophilia, participants were administered an adapted version of the Sexual Opinion Scale [25]. The measure included eight items, half of which were reverse coded, including “the thought of engaging in unusual sex practices is highly arousing” and “it would be emotionally upsetting to me to see someone exposing themselves publicly.” An adapted version of the Self-Control Scale [26] was administered consisting of six items, half of which were reverse scored. Sample items include “I am good at resisting temptation” and “I wish I had more self-discipline.” The erotophilia and self-control scales were shortened in order to enhance survey completion rates, given that participants were not offered compensation for taking part in this study.

To assess sexual history, we included several items adapted from past research. Participants were asked how many male sexual partners they had in the past month, the past three months, and in their entire life [27]. In each question, “sexual partner” was defined as someone with whom they had oral or anal sexual contact. They were also asked how many times they had both receptive and insertive anal intercourse without a condom in the past three months [28]. Finally, participants were asked about their STI history. Specifically, participants were asked how often they get tested for HIV and (separately) how often they get tested for other STIs on a five point scale ranging from *every 3 months or less*, *every 4–6 months*, *every 7–11 months*, *every 1–2 years*, to *every 3+ years*. Participants were also asked whether they have ever been diagnosed with HIV and (separately) whether they have ever been diagnosed with an STI other than HIV. For participants who had been diagnosed with an STI other than HIV, they were given a checklist that allowed them to select which of the following STIs they had tested positive for: chlamydia, gonorrhea, herpes, hepatitis, human papilloma virus (HPV), syphilis, and trichomoniasis. All participants also completed a standard demographics measure that inquired about sexual and gender identity, race, age, nationality, and relationship status [8].

## Results

### Participant Demographics

A total of 252 persons provided consent and began the survey; however, 74 did not answer any questions at all or did not advance far enough in the survey to provide sufficient data for analyses. Data from 66 participants were excluded because they reported that they were having sexual contact only with their current romantic relationship partner. We limited our data only to those who were actively seeking sexual partners to make our comparison groups more equivalent. This resulted in a final sample of 112 individuals, all of whom indicated that they were either currently single (69.6%) or involved in a romantic relationship, but having sexual contact with other persons in addition to their primary partner (30.4%). With respect to gender identity, most identified as male (96.4), with the rest indicating they were transgendered (2.7%) or something else (0.9%). In addition, most participants identified as gay (80.4%), with the remainder identifying as bisexual (11.6%), heterosexual (3.6%), pansexual (3.6%), or something else (0.9%). Self-identified heterosexuals were retained for analyses because our interest was primarily in sexual behavior, not sexual identity. The mean age was 29.97 (*SD* = 10.84; Range = 18 to 63), and the majority of participants (80.4%) were under age 40. Participants were predominately White (86.2%) and from the United States (81.3%).

Participants were roughly evenly divided between those who reported currently having an account on at least one smartphone sex/dating/hookup app (54.5%) and those who did not (45.5%). Analyses were conducted to examine whether app users differed from non-users in terms of their demographic characteristics. Chi-square analyses (for categorical variables) and ANOVAs (for continuous variables) revealed no differences between groups in gender identity, sexual identity, race, age, country of residence, or relationship status (detailed results of these analyses are available from the first author upon request). Thus, the two groups appeared roughly equivalent in terms of their demographic composition. See Table 1 for specific demographic features of the two subsamples.

**Table 1:** Demographic characteristics of app users and non-users.

	App users (n = 61)	Non-users (n = 51)
	Percentage of sample	Percentage of sample
**Identification as male**	96.7%	96.2%
**Sexual Identity**		
Bisexual	9.8%	13.5%
Gay/homosexual	86.9%	73.1%
Heterosexual	1.6%	5.8%
**Identification as White/Caucasian**	86.7%	86.0%
**In a current relationship**	27.9%	32.7%
**Residing in the United States**	80.3%	82.7%
**Age**	30.72 (10.10)	28.87 (11.68)

*Note*. All numbers represent percentages, except for age, which is presented as mean (standard deviation).

### Descriptive Statistics on Smartphone App Use and Sexual Behavior

Among the 61 app users, the vast majority (77%) reported having an account with Grindr. The remainder reported a range of other apps including Adam4Adam, Growlr, Scruff, and Manhunt. Participants reported logging onto these apps an average of 3.03 times per day (*SD* = 3.27) and spending an average of 11.75 minutes engaged with the application each time before closing it or logging out (*SD* = 17.32). Participants were closely divided between those who have their application(s) enabled to send them “push” notifications (44.3%) and those who do not (55.7%).

In terms of sexual behavior, the median number of oral sex partners met through the apps was 4 (*M* = 15.27, *SD* = 30.61), and the median number of anal sex partners was 2 (*M* = 7.53, *SD* = 15.87). Approximately one-third of app users (32.8%) reported that at least one of these sexual encounters had turned into a romantic relationship.

### Hypothesis Tests

For analyses involving normally distributed continuous variables, ANOVAs were used; for non-normally distributed continuous variables, Mann-Whitney tests were used. Chi-square tests were performed on categorical variables.

We first examined whether app users and non-users differed in number of recent sexual partners. Mann-Whitney tests indicated that app users reported significantly more sexual partners in the past three months (*U* = 715.50, *p*<.001, *r* = .45) and the past one month compared to non-users (*U* = 844.00, *p*<.001, *r* = .40). For means, medians, and standard deviations of these items by group, see Table 2.

**Table 2:** Mean and median number of sexual partners and acts of unprotected anal sex among app users and non-users.

	App users		Non-users	
	Median	Mean (SD)	Median	Mean (SD)
**Number of sexual partners**				
Lifetime total	30.00	75.53_a_ (102.97)	7.00	37.22_b_ (85.92)
Past 3 months	3.00	4.84_a_ (5.12)	1.00	1.77_b_ (4.42)
Past 1 month	1.00	2.03_a_ (2.19)	0.00	0.86_b_ (1.61)
**Number of recent instances of unprotected** **anal sex**				
Insertive	0.00	1.28_a_ (6.47)	0.00	2.38_a_ (6.92)
Receptive	0.00	1.30_a_ (3.48)	0.00	4.28_a_ (16.58)

*Note*. SD = standard deviation. Differing subscripts indicate statistically significant within-row mean differences (*p*<.05).

We next examined whether app users and non-users differed in frequency of specific sexual practices and sexual health outcomes. Contrary to hypotheses, Mann-Whitney tests revealed that app users and non-users did not differ in recent frequency of insertive anal intercourse without a condom, (*U* = 1509.50, *p* = .875, *r* = .01), or receptive anal intercourse without a condom, (*U* = 1488.00, *p* = .613, *r* = .05). See Table 2 for medians, means, and standard deviations of these variables. With respect to sexual health history, no differences were observed in frequency of HIV testing (*F* (1, 100) = 0.32, *p = *.571) or frequency of being tested for other STIs (*F* (1, 103) = 1.81, *p = *.182) (see Table 3). App users (3.4%) and non-users (2.0%) did not differ in likelihood of having been diagnosed with HIV, [*X^2^* (1, *N* = 109) = 0.20, *p* = .659]; however, app users (35%) were significantly more likely than non-users (14%) to have been diagnosed with at least one STI other than HIV, [*X^2^* (1, *N* = 109) = 6.34, *p* = .012]. See Table 4 for rates of specific STI diagnoses reported among app users and non-users.

**Table 3:** Mean levels of STI testing and personality characteristics among app users and non-users.

	App users	Non-users
	Mean (SD)	Mean (SD)
**Frequency of HIV testing**	3.04 (1.31)	2.89 (1.27)
**Frequency of testing for other STIs**	3.41 (1.23)	3.09 (1.27)
**Erotophilia**	7.58 (1.01)	7.40 (1.37)
**Sensation seeking**	5.34 (1.58)	5.36 (1.89)
**Self-control**	5.11 (1.34)	5.23 (1.46)

*Note*. SD = standard deviation. Testing frequency variables were assessed on a scale ranging from 1–5, whereas erotophilia, sensation seeking, and self-control were assessed on scales ranging from 1–9. None of the means presented in this table differed significantly from one another within-row.

**Table 4:** Specific STI diagnoses reported by app users and non-users.

	App users	Non-users
	Number who reported a diagnosis of this STI (% of subsample)	Number who reported a diagnosis of this STI (% of subsample)
**Chlamydia**	5 (8.2%)	2 (3.9%)
**Gonorrhea**	3 (4.9%)	1 (2.0%)
**Herpes**	4 (6.6%)	1 (2.0%)
**Hepatitis**	2 (3.3%)	0 (0.0%)
**HIV**	2 (3.3%)	1 (2.0%)
**Human Papilloma Virus (HPV)**	5 (8.2%)	2 (3.9%)
**Syphilis**	1 (1.6%)	1 (2.0%)
**Trichamoniasis**	1 (1.6%)	0 (0.0%)

Next, we found that app users reported significantly more lifetime sex partners than non-users (*U* = 680, *p*<.001, *r* = .47). For medians, means, and standard deviations, see Table 2. We then repeated this analysis, but first, we subtracted the total number of sex partners app users had met specifically though smartphone apps from their lifetime total. In this revised analysis, the difference in number of partners between users and non-users remained statistically significant (*U* = 1020.50, *p* = .004, *r* = .28).

Finally, we tested for psychological differences between app users and non-users. Results revealed no mean differences in erotophilia (*F* (1, 104) = 0.65, *p = *.423), sensation seeking (*F* (1, 104) = 0.00, *p = *.953), or self-control (*F* (1, 103) = 0.17, *p = *.680). Cronbach’s alpha reliabilities for each of the three psychological measures were as follows: sensation seeking (alpha = .74), erotophilia (alpha = .74), and self-control (alpha = .68). For means and standard deviations of these variables by group, see Table 3. Because no differences emerged between groups, we could not test these personality characteristics as mediators of the association between app use and sexual behavior patterns.

## Discussion

In this sample, MSM who used smartphone applications to find casual sex partners had sexual health histories that differed from those participants who utilized other methods for seeking partners. Specifically, app users reported significantly greater numbers of recent sexual partners relative to non-users. In addition, the percentage of app users who reported having been diagnosed with an STI other than HIV was more than twice as high as the percentage of non-users. This difference did not appear to be due to a testing gap, given that frequency of STI testing did not differ between groups. The fact that frequency of STI testing did not differ and there were no differences in frequency of recent instances of UAI suggests that app users were not necessarily engaging in riskier behaviors across the board.

No demographic or psychological differences were observed between app users and non-users on the variables assessed. However, when app users’ lifetime total sex partners was adjusted for partners met specifically through apps, the difference in lifetime partners between users and non-users remained statistically significant. This suggests that there may be a self-selection effect when it comes to app use. Because app users still reported having had more lifetime partners even when that number was adjusted for partners met through apps, it suggests that app users may be more sexually active to begin with. That said, while these findings might appear to lend support to self-selection hypothesis [7], the correlational nature of the data make it impossible to draw any conclusions about cause and effect. Thus, we do not know whether it is the apps that are driving behavior, or if persons who engage in riskier behavior to begin with are simply drawn to the apps, or if perhaps technology and self-selection have a synergistic effect. One plausible explanation is that there may be differences on other, unassessed personality factors that are driving the effects. For example, research has found that some of the Big Five personality traits are related to sexual risk-taking, including extraversion [29] and conscientiousness [30]. Likewise, sexual compulsiveness (which shares a high degree of variance with self-control) is another individual difference trait that has been associated with seeking sexual partners more frequently [31]. Future research would be well served by assessing these and other personality characteristics that could potentially explain why app users seem more prone to sexual risk-taking. Along these same lines, while generalized sensation seeking (which was assessed in the current study) and sexual sensation seeking are very closely associated, future work would benefit from focusing more specifically on sexual sensation seeking because of its stronger association with risky sexual behavior [32]. Another plausible alternative worth testing in future research is that perhaps app users do not have different personality profiles; instead, it may be that app use liberalizes attitudes toward casual sex. In other words, it could be that app use leads to an increase in partner seeking in general, through both electronic and non-electronic means.

The present study also reveals some important insight into the nature of smartphone apps used to arrange casual sex. For one thing, it seems to be the case that these apps serve multiple purposes, both sexual and romantic. Nearly one-third of app-users reported that one of the sex partners they met through their phone turned into a romantic partner. In addition, it is clear that “hooking up” via one of these apps does not mean just one thing, although it appears that oral sex is a more likely activity than anal sex, consistent with other research on the sexual behaviors of MSM [33].

### Strengths and Limitations

This research represents one of the first inquiries into the nature of MSM who find sexual partners through smartphone apps and the sexual health outcomes associated with this emerging technology. Another strength of this research is that it gave due consideration to potential psychological factors that might be associated with use of these apps. Much of the other research considering the link between technology and sexual health has only considered demographic differences between groups [9].

That said, this research is but a preliminary step and has several important limitations. First, the sample size was relatively small and is by no means representative of the broader MSM population. Also, all of our recruitment methods involved the use of electronic media, which limits the generalizability of our results to MSM who consume or have access to electronic media. Moreover, several participants began but did not complete the survey and it is unknown how responders might differ from nonresponders (e.g., perhaps those who did not complete the survey were more erotophobic given that the overall sample leaned erotophilic). It is therefore important for future research to replicate these findings in a larger and more diverse sample. Second, this research only distinguished among app users and non-users and therefore cannot speak to how these apps stack up to other, more specific methods of meeting. For example, is use of a smartphone app different from the use of other technologies for facilitating casual sex (e.g., sex and dating websites)? We suspect that it is, given that smartphones are much more readily accessible at all times than are laptops or computers, not to mention the fact that apps can be enabled to provide instant notifications of message and yield more information on geographic proximity. However, this remains an important avenue to explore in future research. In addition, it would be worth considering other potential differences between app users and non-users. Although there were no demographic or personality differences on the variables assessed in this study, it is entirely possible that some differences do exist that were not assessed here. Finally, as noted above, this research is correlational and does not tell us anything definitive about how smartphone apps of this nature truly affect sexual behavior.

## Conclusions

Study participants who utilized smartphone apps such as Grindr to find sex reported having more sexual partners and had a higher prevalence of STIs compared to MSM who met their partners in other ways. Although no psychological or demographic differences between app users and non-users were observed, we found that app users’ lifetime total sex partners was higher than that of non-users even when we corrected for the number of partners met specifically through smartphone apps. This suggests that there may be some self-selection factors at play in explaining the differential sexual health risks associated with app use; however, the nonrepresentative sample and the nature of the data prevents us from generalizing these findings to MSM at large and from making causal inferences. Although a number of alternative explanations for the observed differences remain possible, the present findings suggest that it is important to give further consideration to the role that smartphone apps play in the sexual health of MSM.
